# Guided Bone Regeneration Effects on Bone Quantity and Outcomes of Dental Implants in Patients With Insufficient Bone Support: A Single-Center Observational Study

**DOI:** 10.7759/cureus.38988

**Published:** 2023-05-14

**Authors:** Eriselda Simoni (Malushi), Renato Isufi, Denis Kadaifciu

**Affiliations:** 1 Oral and Maxillofacial Surgery, University Dental Clinic, Tirana, ALB; 2 Oral Surgery, University Dental Clinic, Tirana, ALB

**Keywords:** dental surgery, dental procedures, insufficient bone support, dental implants, guided bone regeneration

## Abstract

Background

Guided bone regeneration (GBR) is used to influence on stabilization of dental implants in patients with insufficient bone quantity and anatomical problems. But many studies using GBR resulted in divergent results according to the efficiency of new bone quantity formation and implant survival. This research aimed to study the effects of GBR on the increase of bone quantity and short-term stabilization of dental implants in patients with insufficient bone support.

Methodology

The study included 26 patients that underwent the procedure for 40 dental implants from September 2020 to September 2021. In each case, the vertical bone support was intraoperatively measured, through the MEDIDENT Italia paradontal millimetric probe (Medident Italia, Carpi, Italy). The vertical bone defect was considered when the mean vertical depth between the abutment junction and the marginal bone was greater than 1mm up to 8mm. In the group with the presence of the vertical bone defect, GBR technique was used during the procedure of dental implants realized with synthetic bone graft, resorbable membrane, and platelet-rich fibrin (PRF), and the group was considered the study (GBR) group. The group of patients with no vertical bone defects (less than 1mm) and no need for any GBR technique use was considered the control (no-GBR) group. The bone support was evaluated again intraoperatively after six months in both groups when the healing abutments were positioned. The vertical bone defect for each group in baseline and after six months is presented as mean±SD and compared using a t-test. A t-test for Equality of Means was used to calculate the mean depth difference (MDD) between baseline and six months values in each group (GBR and no-GBR) and also between both groups. P-value ≤ 0.05 is considered statistically significant.

Results

Overall 40 dental implants were placed, 20 of them were included in the GBR group and 20 in the no-GBR group. In the GBR group, a statistically significant greater mean vertical bone defect in baseline (day 1), compared to the no-GBR group was found (-4.46±2.76 vs -0.27±0.22; MDD = -4.19 [-5.44 to -2.94] p<0.001). At six months of follow-up in the GBR group, a new bone around the implant was formed, presenting a significantly lower bone defect compared to the baseline measure (-0.39±0.43 vs -4.46±2.76; MDD = -4.07 mm [-5.37 to -2.78] p<0.001). In six months, no statistically significant difference between GBR and no-GBR group in bone support was found (-0.39±0.43 vs -0.27±0.22; MDD = -0.19 [-0.40 to -0.03] p=0.10). In each group, only one implant failure was observed.

Conclusions

The use of GBR showed an important reduction of vertical depth defect between healing abutment and marginal bone predisposing similar short-term stability and survival of dental implants. The use of GBR techniques could be essential in the stabilization of dental implants in patients with insufficient bone support.

## Introduction

A dental implant is a prosthesis that interfaces with the jaw bone or skull to support a dental prosthesis such as a crown, bridge, or denture. The basis for modern dental implants is a biological process called osseointegration, in which materials such as titanium or zirconia form an intimate bond to the bone. The implant fixture is first placed so that it is likely to osseointegrate then a dental prosthesis is added. A variable amount of healing time is required for osseointegration before either the dental prosthesis (a tooth, bridge, or denture) is attached to the implant or an abutment is placed which will hold a dental prosthesis/crown [1].

The stability of dental implants depends on the sufficient quantity and quality of bone formation of the entire surface at bone-to-implant contact and the integration of implants on this osseous tissue [2,3]. Sometimes during the procedure of dental implantation, different techniques to increase the bone volume are used. Successful techniques to treat peri-implant bone defects are the guided bone regeneration (GBR) ones [4-6].

Guided bone regeneration is a dental surgical procedure that uses barrier membranes to direct the growth of new bone and gingival tissue at sites with insufficient volumes or dimensions of bone for proper function, esthetics, or prosthetic restorations. Guided bone regeneration typically refers to ridge augmentation or bone regenerative procedures [7]. This technique allows for the placement of dental implants in areas of insufficient amounts of bone, in localized ridge defects. The PASS principle (primary closure; angiogenesis; space maintenance; stability) is suggested by Wang and Boyapati for a successful GBR [8].

The use of guided bone regeneration techniques performed with bone synthetic graft material, resorbable membrane, and platelet-rich fibrin (PRF) has resulted in studies to have an important effect on the stabilization of dental implants in patients with insufficient bone quantity and anatomical problems [9]. But there are many other studies with divergent results, which reported the loss of marginal bone [10,11], or no change in bone quantity and prognosis in implants with the use of GBR techniques compared to those without GBR [12-14].

The question that arises is if the implants placed in sites associated with bone regeneration provide survival and success rates similar to those placed in sites with sufficient native bone. Our study aims to study the effects of GBR techniques on bone quantity and short-term stabilization and survival of dental implants in patients with insufficient bone support.

## Materials and methods

Patient population

The patient population included 26 patients that underwent the procedure for 40 dental implants from September 2020 to September 2021. The study complies with the Declaration of Helsinki and was approved by the Ethics Committee of the University Dental Clinic (Ethical Act Number: 113). Due to the prospective nature of the study, patients' written informed consent was obtained. The study included all patients over 18 years old and excluded all patients with uncontrolled diabetes mellitus or other systemic diseases, active infectious diseases, or under antibiotic therapy.

The patients were divided into two groups. In the study group (GBR group), there were 14 patients with vertical depth defects who during the procedure of 20 dental implantations received GBR with synthetic bone graft, resorbable membrane, and PRF, meanwhile, 12 patients without vertical depth defects who didn't receive GBR during the procedure of 20 dental implantations were placed in the control group (no-GBR group).

Surgical intervention

The dental implantation procedure was performed by cutting the mucoperiosteal flap and drilling the holes in the position where the dental implants are placed in both groups. In both groups, the initial, primary implant stability was evaluated in vivo and controlled through the insertion torque value. The insertion torque value depends on the individual jaw bone texture, the drilling procedure used, and the type of implant used. We used a minimum initial insertion torque value of 30 Ncm for all types of implants used. The surgeon depending on the case used different techniques to arrive at the primary stability value based on implant size (diameter) and/or the drilling procedure (preparation size).

The implants which were used in our study were "Megagen AnyRidge" (Megagen, Seoul, South Korea). In the control group (no-GBR) the implants used are listed according to the size as follows:

diameter 3.5 mm x length 8 mm (2 implants); 10 mm (5); 11.5 mm (1)

diameter 4 mm x length 7 mm (2); 8.5 mm (2); 11.5 mm (3)

diameter 4.5 mm x length 11.5 mm (1)

diameter 5 mm x length 7 mm (1); 8.5 mm (2); 11.5 mm (1)

In the study group (GBR) the implants were as follows:

diameter 3.5 mm x length 10 mm (2); 11.5 mm (4)

diameter 4 mm x length 7 mm (2); 8.5 mm (2); 10 mm (1); 11.5 mm (1)

diameter 4.5 mm x length 8.5 mm (3); 10 mm (1)

diameter 5 mm x length 7 mm (1); 8.5 mm (1); 10 mm (1); 11.5 mm (1)

The dental stability was also controlled during the first, second, and sixth follow-up months.

Bone defect measurement

**Figure 1:**
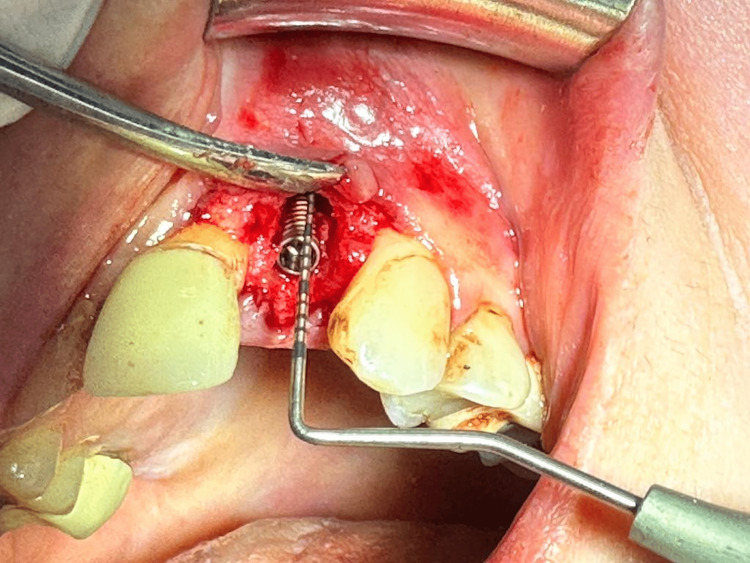
Measurement of defects in vertical bone support through paradontal millimetric probe Labial vertical depth defect 5mm (GBR group).

In each case, the vertical bone support depth was intraoperatively measured between the abutment junction and the marginal bone, through the MEDIDENT Italia paradontal millimetric probe (Medident Italia, Carpi, Italy) (Figure 1). The vertical bone support depth was measured at lingual, medial, buccal and distal sites.

**Figure 2:**
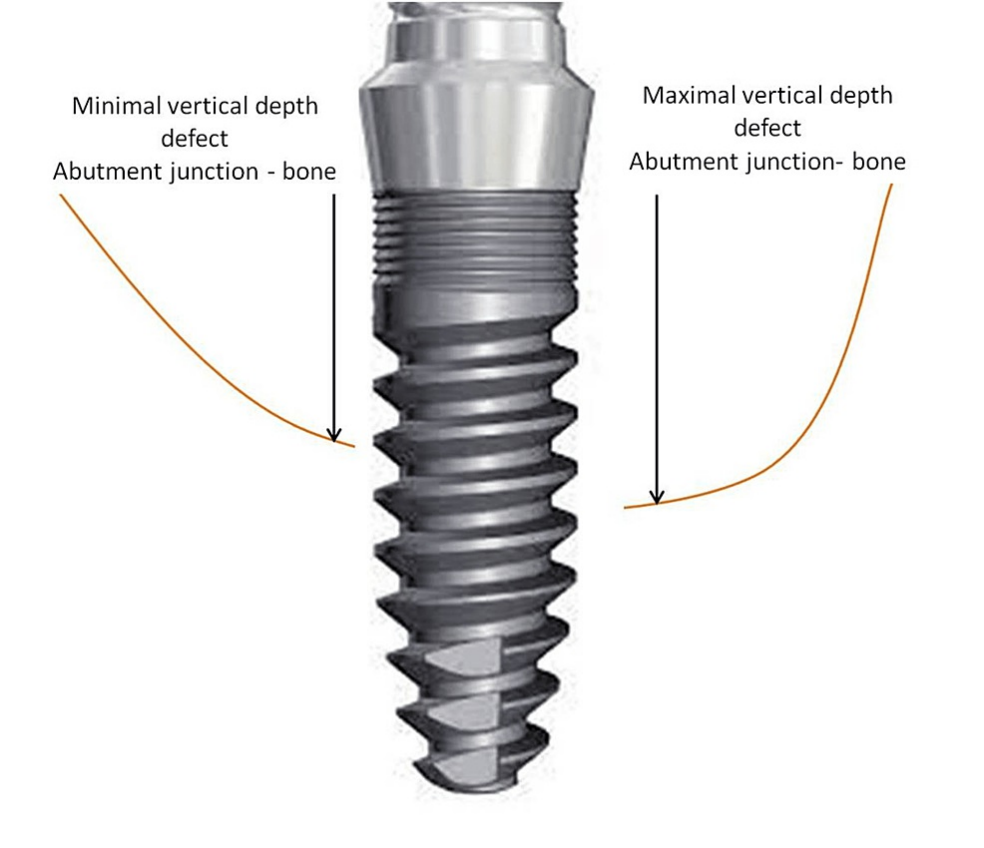
Measurement of vertical bone defect during the dental implantation procedure The vertical bone defect is measured as a mean value of maximal vertical depth and minimal vertical depth defect between the abutment junction and marginal bone.

**Figure 3:**
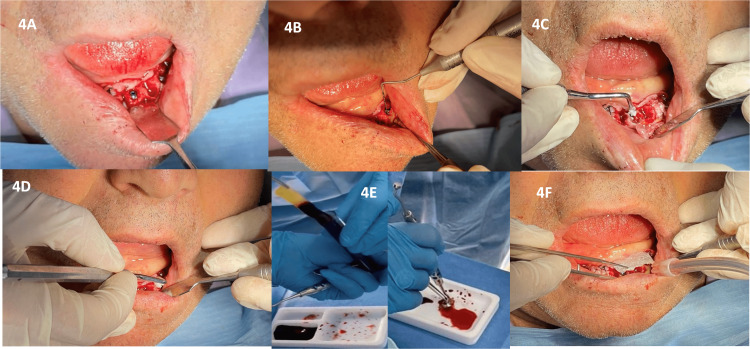
Dental implants placement associated with the use of GBR techniques 4A: Dental implants placement; 4B: Vertical bone defect measurement; 4C and 4D: Synthetic bone graft placement; 4E: Platelet-rich fibrin (PRF) membrane preparing; 4F: PRF and synthetic membrane placement.

**Figure 4:**
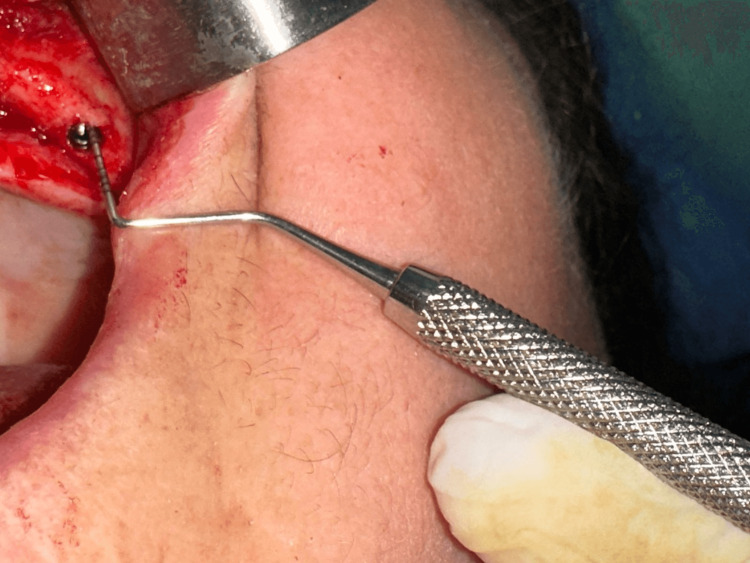
Measurement of vertical bone support through paradontal millimetric probe Vertical bone defect measured less than 1 mm (no-GBR group).

The vertical bone defect is evaluated as a mean value of maximal vertical support depth and minimal vertical depth defect measured. This bone defect evaluation is used previously also from other authors in their research [15]. The vertical bone defect was considered when the mean value of vertical depth between the abutment junction and the marginal bone was greater than 1mm up to 8mm (Figure 2), and these implants were included in the study (GBR) group (Figure 1 and Figure 3). Implants with mean values less than 1mm (Figure 4) were included in the control (no-GBR) group. Implants with a mean vertical bone defect of more than 8mm were not included in the study.

**Figure 5:**
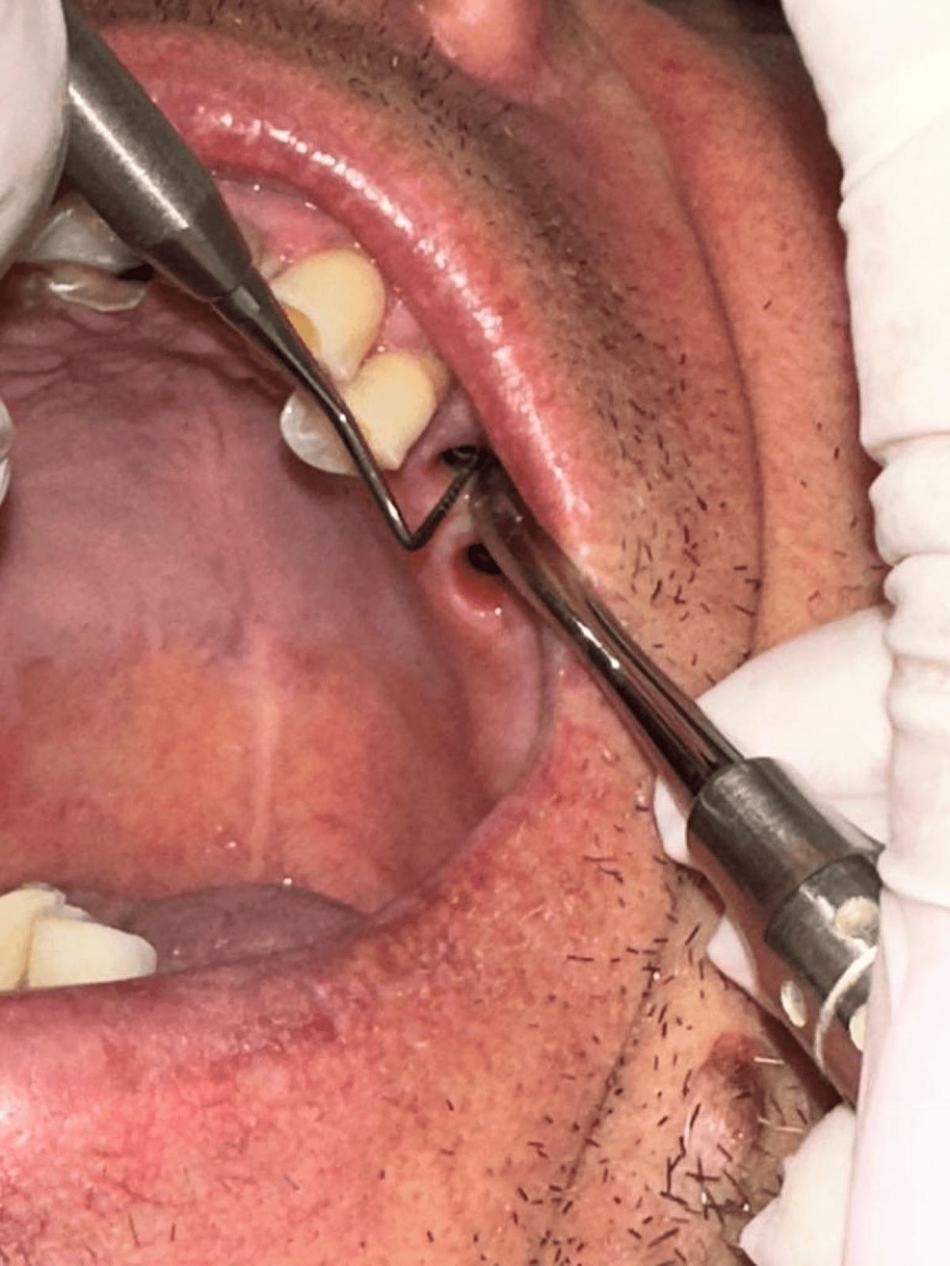
Intraoperatively vertical bone support depth evaluation after six months during the placement of the healing abutments.

**Figure 6:**
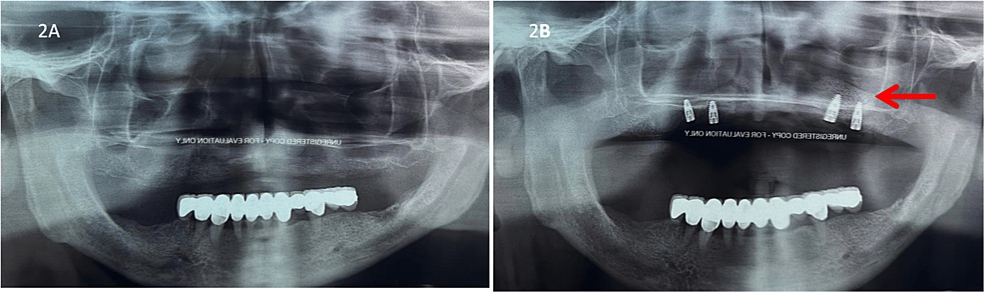
Radiographs for evaluating the bone level 2A: Before the implant placement; 2B: After the implant placement in the GBR group. Arrow showing the new bone level.

The vertical bone support depth was evaluated again intraoperatively after six months in both groups when the healing abutments were positioned (Figure 5). The radiographs were also used to evaluate the bone level before the procedure and after six months of follow-up in each case (Figure 6). Primary endpoints were considered the implant survival and the presence of bone regeneration in the GBR group evaluated by the reduction of the vertical bone defect.

Statistical analysis

Demographic characteristics and procedural continuous data are presented as mean ± SD and compared using the t-test: Discrete data are shown as counts and proportions and are compared using the chi-squared (χ2) test.

A t-test for Equality of Means was used to calculate the mean depth difference (MDD) for evaluating vertical bone support between baseline and six months values in each group (GBR and no-GBR) and also the MDD between both groups. The mean difference between the groups is presented with a 95% confidence interval. P-value <= 0.05 is considered statistically significant. The statistical analysis was performed using the Statistical Package for the Social Sciences Version 21.0 (IBM Corp., Armonk, NY, USA).

## Results

Basal characteristics

Overall there were 40 implants, 20 of them were included in the GBR group and 20 in the no-GBR group. In the GBR group, females were significantly predominant compared to the no-GBR group (70% vs 10%, p<0.001). Meanwhile, no differences in mean age, comorbidities, and implant localization were found (Table 1).

**Table 1:** Basal characteristics of patients undergoing dental implantation procedures GBR: Guided bone regeneration

All implants	GBR 20 patients	no-GBR 20 patients	P value
Gender (female) n (%)	14 (70%)	2 (10%)	<0.001
Age mean (SD)	47.7 ± 10.3	43.35 ± 12.7	0.241
D. Mellitus tp.2 n (%)	1 (5%)	4 (20%)	0.152
Maxilla n (%)	7 (35%)	7 (35%)	N/A
Mandible n (%)	13 (75%)	13 (75%)	N/A

Outcomes of dental implant placements

In the GBR group, a statistically significant greater mean vertical bone defect in baseline (day 1), compared to the no-GBR group was found (4.46±2.76 vs 0.27±0.22; Mean Depth Difference [MDD] -4.19 [-5.44 to -2.94] p<0.001) (Table 2). In the sixth month, no statistically significant difference between GBR and no-GBR group was found (0.39±0.43 vs 0.27±0.22, MDD -0.19 [-0.40 to 0.03] p=0.10). Also, no differences in implant survival were observed between both groups during the six-month follow-up (Table 2). Also as mentioned above, the radiographs were also used to evaluate the bone level before the procedure and after six months of follow-up in each case (Figure 2).

**Table 2:** Intraoperator vertical support and at six months follow-up in GBR and no-GBR groups, mean depth difference between groups, and respective implant survival. GBR: Guided bone regeneration; i.o.: Intraoperatively

All implants	GBR 20 patients	no-GBR 20 patients	Mean Difference, 95% CI	P value
Vertical support i.o.	-4.46±2.76	-0.27±0.22	-4.19 [-5.44 to -2.94]	<0.001
Vertical support 6 months	-0.39±0.43	-0.20±0.18	-0.19 [-0.40 to 0.03]	0.1
Implant survival	19 (95%)	19 (95%)		NA

At six months of follow-up in the GBR group, a new bone around the implant was formed, presenting a significantly lower bone defect compared to the baseline measure (0.39±0.43 vs 4.46±2.76, p<0.001 with a significant reduction of socket depth by a mean of 4.07 mm, p<0.001) (Table 3). No changes in the no-GBR group between baseline measures and six-month follow-ups were observed.

**Table 3:** Mean depth differences in each group (respectively GBR and no-GBR between baseline and six months socket depth) GBR: Guided bone regeneration; MDD: Mean depth difference

Implants	Baseline socket depth	6 months socket depth	MDD	95% CI	P value
GBR group (mm)	-4.46±2.76	-0.39±0.43	-4.07	-5.37 to -2.78	<0.001
no-GBR group (mm)	-0.27±0.22	-0.20±0.18	-0.07	-0.20 to 0.06	0.31

## Discussion

We documented that 1) both implants placed with concomitant GBR and those placed in the native bone had the same survival at six months, 2) an important reduction of vertical depth defect in the GBR group from -4.46±2.76 to -0.27±0.22 with MDD 4.19, p<0.001 resulting in new osseous tissue formation.

This bone regeneration is responsible for the no differences at six months follow-up in socket depths between the two groups (GBR and no-GBR groups, respectively) (0.39±0.43 mm vs 0.20±0.18 mm, MDD -0.19; p=0.10), probably explaining the possible same implant survival rates.

Our results are congruent with other studies conducted previously. Mayfield et al. in 1998 [16] and Zitzmann et al. in 2001 [17] concluded that survival and success rate in placing dental implants have no significant difference between implants in regenerated and non-regenerated bone. Also, Benic et al. [18] in a five-year analysis reported similar survival rates of 100% for the GBR group and 94.1% for the control group without statistical significance. The same authors in a 15-year analysis [19] reported no differences in implant survival respectively 95.6% for GBR and 94.1% for control implants, in interproximal bone levels and dimensions of buccal bone and mucosa. In a systematic review and meta-analysis of eight studies regarding dental implant outcomes in grafted sockets, Ramanauskaite et al. in 2019 [20] reported that implant survival ranged from 95 to 100% for the grafted implants and from 92 to 100% for the non-grafted implants.

Anyway, there are other studies with controversial findings. Huang et al. found more peri-implant crestal bone loss during the healing period in augmented than in pristine bone (0.74 ± 0.74 mm vs. 0.25 ± 0.55 mm) [10]. Also, Mengel et al. found a slightly increased attachment loss (0.65 mm) and bone loss (1.78 mm) that were recorded at the implants in the regenerated bone after three years of loading, but with 100% survival in both groups [10].

The results of our study demonstrate the importance of bone regeneration in patients with insufficient bone support, for the stabilization and survival of dental implants.

Strength and limitations

To our knowledge, this is the first prospective study, analyzing the outcomes of implants placed with or without guided bone regeneration in Albania. Although this is a small study including 26 patients with the placement of 40 implants, it presents the first view, and should be followed by other larger and longer follow-up studies.

## Conclusions

The use of GBR showed an important reduction of vertical depth defect between healing abutment and marginal bone predisposing similar short-term stability and survival of dental implants. The use of GBR could be essential in the stabilization of dental implants in patients with insufficient bone support. Other studies with a greater number of patients and with a longer follow-up are needed for a better evaluation of the effect of GBR techniques on implant stabilization and survival.
